# Specificity of serological screening tests and reference laboratory tests to diagnose gambiense human African trypanosomiasis: a prospective clinical performance study

**DOI:** 10.1186/s40249-024-01220-5

**Published:** 2024-07-08

**Authors:** Martial Kassi N’Djetchi, Oumou Camara, Mathurin Koffi, Mamadou Camara, Dramane Kaba, Jacques Kaboré, Alkali Tall, Brice Rotureau, Lucy Glover, Mélika Barkissa Traoré, Minayegninrin Koné, Bamoro Coulibaly, Guy Pacome Adingra, Aissata Soumah, Mohamed Gassama, Abdoulaye Dansy Camara, Charlie Franck Alfred Compaoré, Aïssata Camara, Salimatou Boiro, Elena Perez Anton, Paul Bessell, Nick Van Reet, Bruno Bucheton, Vincent Jamonneau, Jean-Mathieu Bart, Philippe Solano, Sylvain Biéler, Veerle Lejon

**Affiliations:** 1 Laboratory of Biodiversity and Ecosystem Management, Jean Lorougnon Guédé University, Daloa, Côte d’Ivoire; 2 National Program for Neglected Tropical Disease Control, Patient Management, Ministry of Health, Conakry, Guinea; 3 Trypanosomosis Research Unit, Pierre Richet Institute, Bouaké, Côte d’Ivoire; 4 International Research and Development Centre on Livestock in Subhumid Zones, Bobo-Dioulasso, Burkina Faso; 5 National Program for Malaria Control, Conakry, Guinea; 6 Parasitology Unit, Institut Pasteur de Guinée, Conakry, Guinea; 7 Trypanosome Molecular Biology Unit, Department of Parasites and Insect Vectors, Pasteur Institute, Paris Cité University, Paris, France; 8 Intertryp, IRD-CIRAD-University of Montpellier, Montpellier, France; 9 Independent Consultant, Edinburgh, UK; 10 Department of Biomedical Sciences, Institute of Tropical Medicine, Antwerp, Belgium; 11 Foundation for Innovative New Diagnostics, Geneva, Switzerland

**Keywords:** Human African trypanosomiasis, *Trypanosoma brucei gambiense*, Diagnosis, Specificity, Rapid diagnostic test, Immunological test, Molecular test

## Abstract

**Background:**

Serological screening tests play a crucial role to diagnose gambiense human African trypanosomiasis (gHAT). Presently, they preselect individuals for microscopic confirmation, but in future “screen and treat” strategies they will identify individuals for treatment. Variability in reported specificities, the development of new rapid diagnostic tests (RDT) and the hypothesis that malaria infection may decrease RDT specificity led us to evaluate the specificity of 5 gHAT screening tests.

**Methods:**

During active screening, venous blood samples from 1095 individuals from Côte d’Ivoire and Guinea were tested consecutively with commercial (CATT, HAT Sero-*K*-SeT, Abbott Bioline HAT 2.0) and prototype (DCN HAT RDT, HAT Sero-*K*-SeT 2.0) gHAT screening tests and with a malaria RDT. Individuals with ≥ 1 positive gHAT screening test underwent microscopy and further immunological (trypanolysis with *T.b. gambiense* LiTat 1.3, 1.5 and 1.6; indirect ELISA/*T.b. gambiense*; *T.b. gambiense* inhibition ELISA with *T.b. gambiense* LiTat 1.3 and 1.5 VSG) and molecular reference laboratory tests (PCR TBRN3, 18S and TgsGP; SHERLOCK 18S Tids, 7SL *Zoon*, and TgsGP; *Trypanozoon* S^2^-RT-qPCR 18S2, 177T, GPI-PLC and TgsGP in multiplex*;* RT-qPCR DT8, DT9 and TgsGP in multiplex). Microscopic trypanosome detection confirmed gHAT, while other individuals were considered gHAT free. Differences in fractions between groups were assessed by Chi square and differences in specificity between 2 tests on the same individuals by McNemar.

**Results:**

One gHAT case was diagnosed. Overall test specificities (*n* = 1094) were: CATT 98.9% (95% *CI*: 98.1–99.4%); HAT Sero-*K*-SeT 86.7% (95% *CI*: 84.5–88.5%); Bioline HAT 2.0 82.1% (95% *CI*: 79.7–84.2%); DCN HAT RDT 78.2% (95% *CI*: 75.7–80.6%); and HAT Sero-*K*-SeT 2.0 78.4% (95% *CI*: 75.9–80.8%). In malaria positives, gHAT screening tests appeared less specific, but the difference was significant only in Guinea for Abbott Bioline HAT 2.0 (*P* = 0.03) and HAT Sero-*K*-Set 2.0 (*P* = 0.0006). The specificities of immunological and molecular laboratory tests in gHAT seropositives were 98.7–100% (*n* = 399) and 93.0–100% (*n* = 302), respectively. Among 44 reference laboratory test positives, only the confirmed gHAT patient and one screening test seropositive combined immunological and molecular reference laboratory test positivity.

**Conclusions:**

Although a minor effect of malaria cannot be excluded, gHAT RDT specificities are far below the 95% minimal specificity stipulated by the WHO target product profile for a simple diagnostic tool to identify individuals eligible for treatment. Unless specificity is improved, an RDT-based “screen and treat” strategy would result in massive overtreatment. In view of their inconsistent results, additional comparative evaluations of the diagnostic performance of reference laboratory tests are indicated for better identifying, among screening test positives, those at increased suspicion for gHAT.

**Trial registration:**

The trial was retrospectively registered under NCT05466630 in clinicaltrials.gov on July 15 2022.

**Graphical Abstract:**

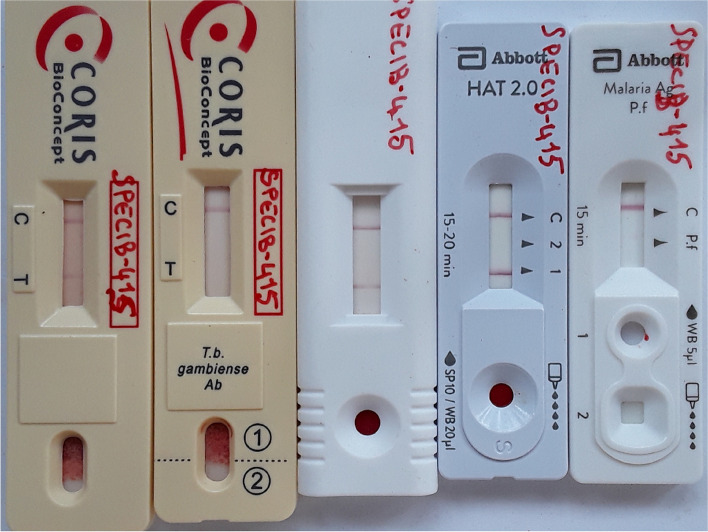

**Supplementary Information:**

The online version contains supplementary material available at 10.1186/s40249-024-01220-5.

## Background

Gambiense human African trypanosomiasis (gHAT) is caused by infection with the parasite *Trypanosoma brucei gambiense (T.b. gambiense)*, which is transmitted by tsetse flies in West and Central Africa. The disease has been targeted by the World Health Organization (WHO) for elimination as a public health problem by 2020 and as zero transmission by 2030 [1, 2]. Elimination as a public health problem has been validated so far for 7 countries, including Côte d’Ivoire, and is being reached by others, including Guinea [3, 4]. To reach gHAT elimination, continued surveillance is required, which strongly relies on serological screening of the population at risk. At present, parasitological confirmation of screening test seropositives is usually required before gHAT treatment is administered [5]. However, the availability of a safe, easy-to-use drug in the near future would render a “screen and treat” strategy -without need for parasitological confirmation of seropositive individuals- a realistic strategy to accelerate elimination, given the availability of simple and accurate diagnostic screening tests [6].

Different commercialized and prototype tests exist for population screening, which all detect trypanosome specific antibodies. The card agglutination test for trypanosomiasis (CATT), was developed 50 years ago and is based on macroscopic agglutination of fixed and stained whole *T.b. gambiense* trypanosomes by specific antibodies [7]. The CATT test is used for active mass screening but is not suitable for individual or passive screening [8]. Individual rapid diagnostic tests (RDT) for gHAT were introduced a decade ago, and two RDTs are currently commercially available. The HAT Sero-*K*-Set (Coris BioConcept, Belgium), is a first generation RDT based on native purified *T.b. gambiense* variable surface glycoprotein (VSG) antigens, Lille Trypanosome antigen type (LiTat) 1.3 and LiTat 1.5, in a single test line [9]. The Abbott Bioline HAT 2.0 (Abbott, Republic of Korea) is a 2nd generation RDT which was commercialized in 2021, and contains recombinant invariable surface glycoprotein 65 (ISG65) and recombinant LiTat 1.5 VSG as antigens, in separate test lines [10]. Two additional second generation RDT prototypes have recently been developed: a DCN HAT RDT prototype (DCN, USA) with the same format as the Abbott Bioline HAT 2.0 RDT, and HAT Sero-*K*-Set 2.0 (Coris BioConcept, Belgium), an RDT containing 3 recombinant antigens -LiTat 1.3 VSG, LiTat 1.5 VSG and ISG65- in a single test line. Although the clinical performance of CATT has been extensively evaluated, prospective comparative evaluations of the diagnostic performance of gHAT RDTs are few or missing, in particular for the 2nd generation RDTs. The influence of other endemic infections, such as malaria, on RDT specificity, has hardly been studied.

To overcome the limited positive predictive values of the gHAT screening tests at low prevalence on the one hand, and the limited sensitivity of the parasitological confirmation tests on the other hand, additional reference laboratory tests can be carried out on screening test positives in whom no trypanosomes were found microscopically or who did not undergo microscopic examination [11]. Owing to the limited specificity of screening tests, there will be potentially large numbers of samples progressing for these additional tests. For this purpose, antibody detection immunological laboratory tests are commonly applied, trypanolysis against *T.b. gambiense* variable antigen type (VAT) LiTat 1.3, 1.5 and/or 1.6 and indirect ELISA/*T.b. gambiense* [12, 13]. Attributed to its comparable clinical specificity [14], the *T.b. gambiense* inhibition ELISA (*g*-iELISA) against *T.b. gambiense* LiTat 1.3 and 1.5 VSG has recently been proposed as a potential replacement for trypanolysis, as the latter can only be performed in selected reference laboratories. Different new molecular test formats have recently been developed. The Specific High Sensitivity Enzymatic Reporter unLOCKing (SHERLOCK) method detects trypanosome RNA based on isothermal recombinase polymerase amplification (RPA), followed by a Cas13-CRISPR RNA recognition targeting either Trypanosomatid, *Trypanozoon* or *T.b. gambiense* specific sequences [15]. The *Trypanozoon*-S^2^-RT-qPCR (reverse transcriptase quantitative polymerase chain reaction) multiplex assay detects four targets in a single reaction, with each target chosen for its particular sensitivity and specificity (S^2^). Two targets, the multicopy minichromosomal *Trypanosoma brucei* repeat (TBR) sequences (177T) [16] and the multicopy 18S rRNA transcripts (18S2), are included to enhance sensitivity, providing specificity limited to *Trypanozoon*. To refine the assay’s analytical specificity, *T.b. gambiense* specific glycoprotein (TgsGP), specific to *T.b. gambiense* is targeted, while single-copy detection in the specimen is demonstrated through targeting the *Trypanozoon*-specific glycosylphosphatidylinositol-specific phospholipase C (GPI-PLC). Finally, RT-qPCR targets *Trypanozoon* DT8, DT9 or *T.b. gambiense* specific TgsGP nucleic acid.

In view of their importance in gHAT management, and even more once the “screen & treat” strategy is introduced, further assessment of the diagnostic performance of these immunological and molecular reference laboratory tests in gHAT seropositives is required [11].

We carried out a prospective clinical performance study on specificity of serological tests for human African trypanosomiasis in Côte d’Ivoire and in Guinea, two gHAT endemic countries with different epidemiological features [3, 4, 17]. The primary objective was to comparatively assess the specificity of five serological screening tests for gHAT- comprising CATT and the 4 RDTs. The secondary objectives were to compare specificities of the five screening tests for gHAT in malaria positive and malaria negative individuals; and to assess the specificity of the immunological and molecular reference laboratory tests in screening test positives. Taking into account the actual low gHAT prevalence in Guinea and in Côte d’Ivoire, we did not attempt to assess the diagnostic sensitivity of the tests.

## Methods

### Prospective clinical performance study set up

The number of participants to be included in SpeSerTryp was calculated based on estimates of test specificity reported in the literature [9, 10] or provided by the serological screening test manufacturers. The mean of the specificity estimates, 99.0%, was used as the reference value for the specificity, the significance level (alpha) was set at 5%, the power at 80% and the equivalence margin at 2% (based on the range of 97.2–99.2% of specificities previously observed with CATT [9, 10]). The minimum sample size required for the comparisons was 424 non-HAT affected controls [18]. Due to the uncertainty of the specificity estimates and to improve precision, it was proposed to include a minimum of 500 non-HAT affected controls in each country.

Inclusions took place consecutively, during active screening by experienced mobile teams between June 21 and July 5, 2022 in the Bonon and Sinfra hypoendemic foci, located in the Bouaflé and Sinfra health districts in Central West Côte d’Ivoire [4], and from June 28 to July 2, 2022 in the under-prefectures Ouassou and Khorira, which both can be considered hypoendemic, and are located in Dubréka prefecture in Guinea [3].

Inclusion criteria were being aged 10 years or more and having provided written informed consent for participation. Exclusion criteria were known severe anemia preventing blood collection; severe medical condition preventing the collection of informed consent and participation in the study (e.g. coma, cognitive impairment, etc.) or having a known history of previously treated HAT infection.

An overview of all tests for diagnosis of gHAT that were carried out in the SpeSerTryp study is given in Table 1.

**Table 1:** Overview of diagnostic tests for gambiense human African trypanosomiasis carried out in the SpeSerTryp study

Test	Target	Reference
Field tests		
Commercial gHAT serological screening tests		
CATT	Specific antibodies	[7]
HAT Sero-*K*-Set	Specific antibodies	[9]
Abbott Bioline HAT 2.0	Specific antibodies	[10]
Prototype gHAT serological screening tests		
DCN HAT RDT	Specific antibodies	-
HAT Sero-*K*-Set 2.0	Specific antibodies	-
Reference laboratory tests^a^		
Immunological reference laboratory tests		
Trypanolysis *T.b. gambiense* LiTat 1.3	Specific antibodies	[12, 19]
Trypanolysis *T.b. gambiense* LiTat 1.5	Specific antibodies	[12, 19]
Trypanolysis *T.b. gambiense* LiTat 1.6	Specific antibodies	[12, 19]
Indirect ELISA/*T.b. gambiense*	Specific antibodies	[13, 20]
*g*-iELISA LiTat 1.3^b^	Specific antibodies	[14]
*g*-iELISA LiTat 1.5^b^	Specific antibodies	[14]
Molecular reference laboratory tests^c^		
TBRN3 PCR	*Trypanozoon* DNA	[21]
18S PCR	*Trypanozoon* DNA	[22]
TgsGP PCR	*T.b. gambiense* DNA	[23]
SHERLOCK 18S Tids	Trypanosomatid nucleic acids	
SHERLOCK 7SL *Zoon*	*Trypanozoon* nucleic acids	[15]
SHERLOCK TgsGP	*T.b. gambiense* nucleic acids	[15]
*Trypanozoon*-S2-RT-qPCR 18S2 (in multiplex)	*Trypanozoon* nucleic acids	-
*Trypanozoon*-S2-RT-qPCR 177T (in multiplex)	*Trypanozoon* DNA	[16]
*Trypanozoon*-S2-RT-qPCR GPI-PLC (in multiplex)	*Trypanozoon* nucleic acids	[16]
*Trypanozoon*-S2-RT-qPCR TgsGP (in multiplex)	*T.b. gambiense* nucleic acids	-
RT-qPCR DT8 (in multiplex)	*Trypanozoon* nucleic acids	-
RT-qPCR DT9 (in multiplex)	*Trypanozoon* nucleic acids	-
RT-qPCR TgsGP (in multiplex)	*T.b. gambiense* nucleic acids	-

Parasitology was used as a composite reference standard

*gHAT* Gambiense human African trypanosomiasis

^a^Reference laboratory tests were carried out only when a participant tested positive in one or more serological screening tests

^b^With the internal controls falling out of the specified range, results were considered invalid and are not presented

^c^SHERLOCK RP was run as an extraction control and is not considered as a gHAT diagnostic test

### Tests performed in the field, specimen collection and case management

**Fig. 1:**
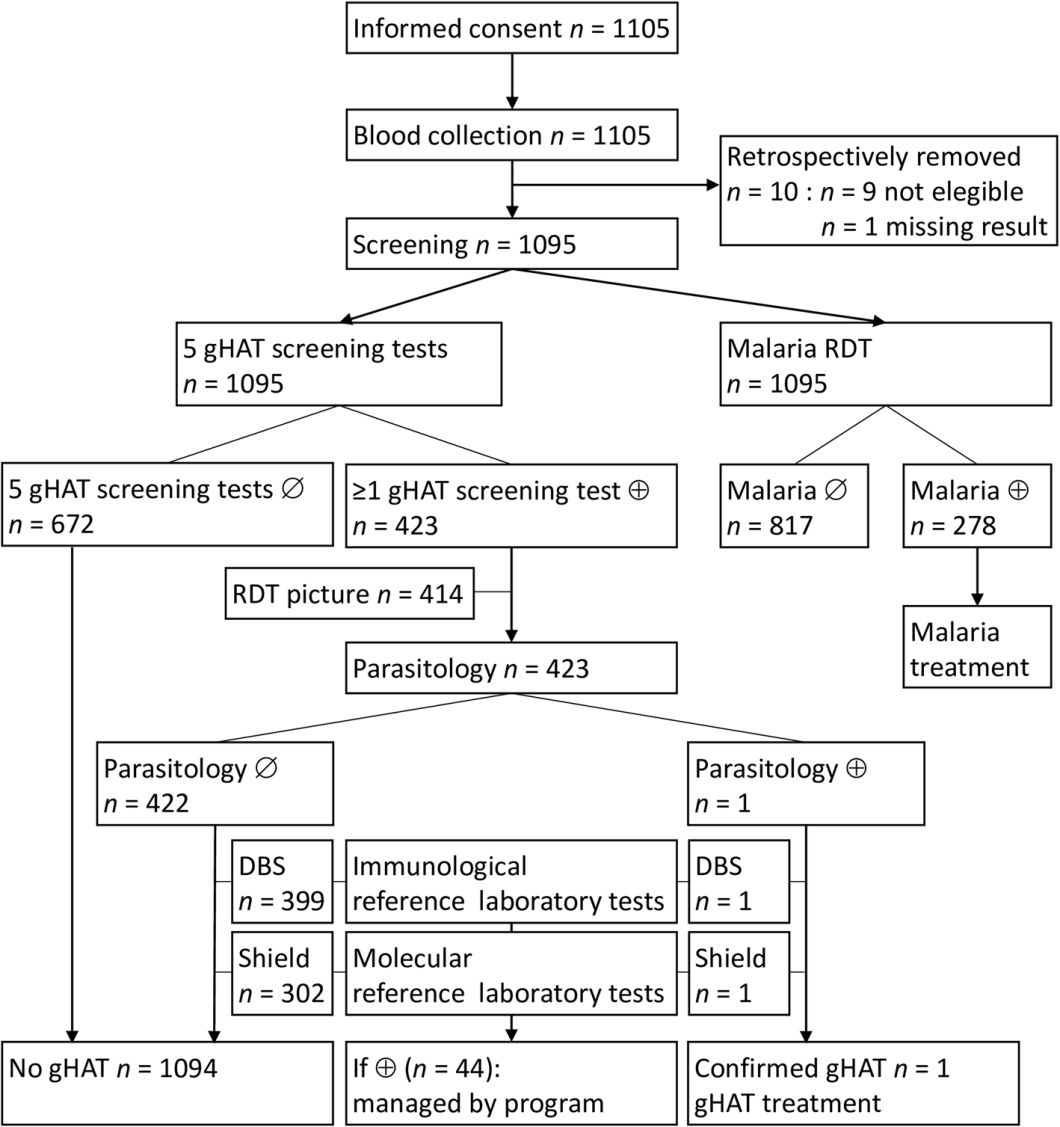
Summary of the test algorithm and data interpretation. *gHAT* gambiense Human African trypanosomiasis; *RDT* rapid diagnostic test; ∅ negative test result; ⊕ positive test result; *DBS* dried blood spot; Shield: blood in DNA/RNA shield buffer

The testing algorithm is summarized in Fig. 1. After having obtained informed consent from each participant or their legal representatives for children under 18 years, 6 mls of blood were collected in a heparinized tube by venepuncture. The heparinized blood was used for all the tests.

The gHAT serological screening tests CATT (Institute of Tropical Medicine, Antwerp, Belgium), Abbott Bioline HAT 2.0 (Abbott, Seoul, Republic of Korea), DCN HAT prototype (DCN, Carlsbad, USA), HAT Sero-*K*-Set (Coris BioConcept, Gembloux, Belgium), and HAT Sero-*K*-Set 2.0 prototype (Coris BioConcept, Gembloux, Belgium) were carried out in parallel, according to the instructions of the manufacturers. Diagnosis of malaria was performed with the Bioline Malaria Ag *P.f.* RDT (Abbott, Seoul, Republic of Korea). If one or more of the 5 gHAT serological screening tests was positive, a picture was taken of the RDT results, parasitological examination was performed (initiated within 1 h of blood collection) and a blood specimen was prepared for ulterior immunological and molecular reference laboratory tests.

For parasitological examination (reference test), 500 µl of heparinised blood was examined in the mini anion exchange centrifugation technique (mAECT) [24]. The mAECT column retains the blood cells, while trypanosomes, if present, flow through. The eluate is then collected in a special collector tube, which was centrifuged at 1500 × *g* for 10 min. The point of the collector tube was examined directly under the microscope at 100 × magnification for presence of trypanosomes. If mAECT was negative and palpable cervical lymph nodes were present, lymph was collected by lymph node puncture and a drop examined directly under the microscope at 400 × magnification. In the exceptional case of strong clinical suspicion, and if previous parasitological tests on blood and lymph remained negative, a lumbar puncture could be performed. In that case, cerebrospinal fluid was examined for the presence of trypanosomes in the modified simple centrifugation, and the white blood cell number was determined.

Dried blood spots (DBS) for the immunological laboratory tests were prepared by applying 12 spots of 30 µl of blood on Whatman grade 4 paper. The DBS were left to dry in the shadow and after drying, were packed individually in an envelope. Envelopes were packed by 10 in a plastic zip lock bag, to which 35 g of dry silicagel was added, after which the bag was closed hermetically. Specimens for molecular laboratory testing were prepared by mixing 1 ml of heparinized blood with 1 ml of DNA/RNA Shield 2 × buffer (Zymo Research, Irvine, USA). Tubes were frozen at -20 °C within 12 h and next stored at -80 °C.

Diagnosed HAT and malaria cases were treated by national programs according to the routine procedures in place in the country.

### Retrospective scoring of HAT RDT test line intensities

For screening test positives, the intensity of the test lines of the gHAT RDTs was scored retrospectively based on the pictures taken in the field. Two readers, unaware of the field screening test results, independently scored all RDT test line intensities between zero (negative) and 4 (strongly positive) on the picture by comparison with a reference card [25]. If both readers had scored a line intensity the same, this score was retained. When both readers scored a line intensity differently, a third reader –unaware of previous scores- scored the intensity of all gHAT RDT lines on the picture of that seropositive again, and the final score became the intensity score given by 2 out of 3 readers, or, if 3 different scores had been given, the median of the 3 intensities.

### Tests performed in the reference laboratory

#### Immunological reference laboratory tests

Trypanolysis was carried out as previously described, using *T.b. gambiense* variant antigen type LiTat 1.3, LiTat 1.5 and LiTat 1.6 [19]. Trypanolysis was considered positive if the test specimen caused 50% lysis or more. The indirect ELISA/*T.b. gambiense*, using as antigens a mixture of LiTat 1.3 and 1.5 VSG, was carried out according to the standard protocol [13, 20]. Indirect ELISA/*T.b. gambiense* was considered positive if the percent positivity was 30% or more. The *g*-iELISA (apDia, Turnhout, Belgium) was carried out on LiTat 1.3 and on LiTat 1.5 VSG, according to the instructions of the manufacturer [14]. As test samples for the *g*-iELISA, 8 discs of 6 mm diameter were punched out from the DBS and eluted overnight in 400 μl of *g*-iELISA sample diluent. The specimen was considered positive in *g*-iELISA if the percent inhibition was ≥ 30%.

#### Molecular reference laboratory tests

##### Total nucleic acids (TNA) extraction

TNA were extracted from 300 µl of human blood in DNA/RNA 2 × Shield buffer (Zymo Research, Irvine, USA) with the Maxwell RSC DNA blood kit (Promega, Madison, USA) in the Maxwell RSC 16 automated system (Promega, Madison, USA), according to the manufacturer’s instructions. Purified TNA 40 µl aliquots were stored at -80°C.

##### PCR

All DNA extracts were analysed with the TBRN3 and 18S PCRs (Table 2), targeting *Trypanozoon* specific regions, and TgsGP PCR targeting the *T.b. gambiense*-specific glycoprotein as previously described with slight modifications [21–23]. A total volume of 25 μl, 5 μl TNA extract plus 20 μl of PCR mix (1 × Go *Taq* Green Master Mix, 0.5 µmol/L of primer in nuclease-free water), was used for amplification. Amplification was carried out in a SimpliAmp™ thermal cycler (Thermo Fisher Scientific, Waltham, USA) with the following parameters: an initial cycle of denaturation at 95 °C for 5 min, then 35 cycles consisting of denaturation at 95 °C for 45 s, hybridization at 50 °C (TBRN3 PCR) or 60 °C (18S and TgsGP PCR) for 45 s, and elongation at 72 °C for 45 s, and finally a last elongation cycle at 72°C for 5 min. Amplicons were separated by electrophoresis in 2% agarose gel stained with gelRed for 45 min at 100 V. The ChemiDoc TM imaging system (Bio-Rad, Hercules, USA) was used to visualize and record results. Fragment sizes were checked using the GeneRuler Ladder size marker (Thermo Fisher Scientific, Waltham, USA).

**Table 2:** PCR target genes and primers used in the study

PCR	Target gene (accession number)	Primer name	Sequence, 5′ → 3′	Amplicon size
TBRN3	TBR (K00392.1)	TBRN3-F	TAAATGGTTCTTATACGAATGA	168 bp
		TBRN3-R	TTGCACACATTAAACACTAAAGAACA	
18S	18S rRNA (TB927_01.rRNA.1)	M18S-II-F-Tb	CGTAGTTGAACTGTGGGCCACGT	149 bp
		M18S-II-R-Tb	ATGCATGACATGCGTGAAAGTGAG	
TgsGP	TgsGP (FN555988.1)	TgsGP-F	GCTGCTGTGTTCGGAGAGC	308 bp
		TgsGP-R	GCCATCGTGCTTGCCGCTC	

##### SHERLOCK assays

The SHERLOCK assays were carried out as previously described [15]. Briefly, the RPA was performed with TwistAmp Basic kits (TwistDx, Maidenhead, UK) on the purified TNA using reverse transcriptase Transcriptor (Roche, Basel, Switzerland) and RPA primers specific for each target: 18S rRNA (*T.b.* 18S ribosomal RNA), 7SL (*T.b.* 7 spliced-leader RNA), TgsGP (*T.b. gambiense* specific glycoprotein) and RP (human RNase P POP7) (Table 3). The reactions were run for 45 min at 42°C in a heating block.

**Table 3:** SHERLOCK target genes, RPA primer regions and sequences, and DNA IVT region and sequence used for production of the crRNA guide

SHERLOCK	Target gene (accession number)		Name	Region, bp	Sequence (5′ → 3′) (T7 promotor + primer) or (spacer + *DR* + T7 Promoter)
18S Tids	18S rRNA (TB927_01.rRNA.1)	RPA primers	18S P-tryds 1 F	1535–1559	GAAATTAATACGACTCACTATAGGGTTTAATTTGACTCAACACGGGGAAC
			18S P-tryds 1 R	1672–1648	GGAATCAACCAAACAAATCACTCCA
		DNA IVT template	18S P-tryds 3	1620–1647	ATGGTGGTGCATGGCCGCTTTTGGTCGG
7SL *Zoon*	7SLRNA (M80262)	RPA primers	7SLbUP F	109–133	GAAATTAATACGACTCACTATAGGGCGGAGCGCATTGCTCTGTAACCTTC
			7SLb3 R	266–243	CCACTTTAACGGCGCGAGAACGCC
		DNA IVT template	cr7SLb3	197–224	TGTTCTGCTTGGTTGCGTGTCGGTGTTG
TgSGP	TgSGP (FN555988.1)	RPA primers	SGP2 F	437–466	GAAATTAATACGACTCACTATAGGGTTTGACAGCATGGGAGATGCAACTCGCAAG
			SGP2 R	551–580	CAAGTCCGTGACAGCCTTGCCCGTTCCCGC
		DNA IVT template	crSGP2.1	467–494	CTAGCACAGCGGAAGCTGGAAGCCATTT
RP	RNase P POP7 (NM_005837.3)	RPA primers	RP 1F	516–540	GAAATTAATACGACTCACTATAGGGTTGATGAGCTGGAGCCAGAGACCGA
			RP 1R	634–663	CGAAGAGCCATATCACGGAGGGGATAAGTG
		DNA IVT template	crRP1	541–568	CACACGGGAGCCACTGACTCGGATCCGC

*SHERLOCK* Specific High Sensitivity Enzymatic Reporter unlocking, *RPA* Recombinase polymerase amplification, *IVT* In vitro transcription

Specific crRNA guides were prepared by an in vitro transcription (IVT) step with a T7 RNA polymerase (Biosearch technology, Teddington, UK): specific DNA templates (Table 3) were in vitro transcribed using the HiScribe™ T7 Quick High Yield RNA Synthesis Kit (NEB, Ipswich, USA) and purified using magnetic beads (Agencourt RNAClean XP, Beckman, Brea, USA).

The detection step was carried out as previously described [15] using a purified home-made recombinant LwCas13a and the specific crRNA guides prepared as described above. The detection was run in triplicate in 384-well black-plates, F-bottom, μClearbottom (Greiner, Kremsmünster, Austria) at 37 °C in a plate reader (INFINITE F200 PRO Option Infinite F Nano + , TECAN, Männedorf Switzerland). Fluorescence values were recorded at initial time point and after 3 h of incubation.

The SHERLOCK RP assay was carried out first, to validate the quality of the TNA extractions from each sample, before testing the other targets. All reactions included a negative template control (NTC) with nuclease-free water as input, and a positive template control with in vitro transcribed target fragments specific of each SHERLOCK assay (18S Tids, 7SL *Zoon*, TgSGP) as well as TNA from *T.b. brucei* AnTat1.1E or *T.b. gambiense* ELIANE strain. For each sample, the fold-change over the NTC background fluorescence (FC) was calculated by dividing the sample by the NTC fluorescence value at 3 h. Thresholds for positivity were at FC ≥ 7.1 for RP, FC > 5 for 18S Tids, FC > 2.6 for 7SL *Zoon* and FC > 2.1 for TgsGP.

##### Trypanozoon-S^2^-RT-qPCRs

The *Trypanozoon*-S^2^-RT-qPCR reactions were run with 1 × qScript XLT ToughMix (Quantabio, Beverly, USA), using 300 nmol/L primers and 100 nmol/L of fluorescent labelled probes (LGC Biosearch Technologies, Hoddesdon, UK) for each of the four sets (Table 4). Conducted in a 20 µl volume using 5 µl of TNA extract, amplification occurred on a Q-qPCR magnetic induction cycler (Quantabio, Beverly, USA) at 95 °C denaturation for 10 min, followed by 40 cycles of 95 °C for 15 s and 60 °C for 60 s. Amplification analysis was performed using Q-qPCR 1.0.2 software (Quantabio, Beverly USA) using the dynamic method on automatic threshold settings. Prior analysis suggested a specificity cut-off for the *Trypanozoon*-S^2^-RT-qPCR 177T at 30 Cq, and 35 Cq for the *Trypanozoon*-S^2^-RT-qPCRs 18S2, GPI-PLC, and TgsGP.

**Table 4:** *Trypanozoon*-S^2^-RT-qPCR target genes, primers and probes

*Trypanozoon*-S^2^-RT-qPCR	Target gene (accession number)	Fluorophore	Probe sequence	Quencher	Primer F sequence, 5′ → 3′	Primer R sequence, 5′ → 3′
177T	TBR (K00392.1)	FAM	TGCCATATTAATTACAAGTGTGC	BHQ-1 plus	CGCAGTTAACGCTATTATACA	GGACCATTAAATAGCTTTGTTG
18S2	18S rRNA (XR_002989995.1)	CAL Fluor Orange 560	TTGTGTTTACGCACTTGTCGTGGC	BHQ-1 plus	CCAATCGGACGCTCTCTTT	GTGGAGGCGTTGGTTCTAAT
GPI-PLC	GPI-PLC (X13292.1)	CAL Fluor Red 610	ACACCACTTTGTAACCTCTGGCAGT	BHQ-1 plus	CCCACAACCGTCTCTTTAACC	GGAGTCGTGCATAAGGGTATTC
TgsGP	TgSGP (FN555988.1)	Quasar 670	CTCTCCGAACACAGCAGCGACATC	BHQ-2 plus	GAAGCAGTGGGACCTTAGC	TTTGTGCTCTTGCTTGCTATTAC

##### RT-qPCRs DT8, DT9 and TgsGP

Probes were defined from Tb927.10.8530 (DT8) and Tb927.10.1090 (DT9). For DT8, forward 5’-GCTTCTCCCGTTGATGTC-3’ and reverse 5’- AATATCGGTTACGTCGCC-3’ primers amplified a 212 bp fragment with HEX-CTCGCTCGCATGACTCAT-BHQ-1 as probe. For DT9, forward 5’-AACCCCTGGAGGACATC-3’ and reverse 5’-GCTTTGTACCGTCAGAAGA-3’ primers amplified a 144 bp fragment with FAM- CCGTGGTTGAATAGTGAACCG-BHQ-1 as probe. A third primers/probe set targeted the TgsGP gene as previously described [26]. The multiplex RT-qPCR conditions were as follows: 200 nmol/L primers and 400 nmol/L of fluorescent labelled probes (LGC Biosearch Technologies, Hoddesdon, UK) for each of the three sets were mixed with 1 × qScript XLT ToughMix (Quantabio, Beverly, USA) and 5 µl TNA extract in a 20 µl final volume. In a Q-qPCR magnetic induction cycler (Quantabio, Beverly, USA), after a 50 °C reverse transcription step for 10 min, followed by a 95 °C denaturation step for 10 min, amplification was performed by 40 cycles of 95 °C for 15 s and 60 °C for 60 s. Amplification analysis was performed using Q-qPCR 1.0.2 software (Quantabio, Beverly, USA) using the dynamic method on automatic threshold settings. Prior analysis suggested a specificity cut-off for the RT-qPCR DT8, DT9 and TgsGP sets at 35 Cq.

### Data analysis

For the analysis of the study results, data from individuals who were not eligible but were included by accident, or with missing field test results (RDT or parasitology not carried out) were removed from the dataset. Test results, with the exception of gHAT RDT line intensities, were transformed into qualitative results in order to construct contingency tables. A participant was defined as a gHAT case if the presence of trypanosomes was demonstrated microscopically in lymph, blood or —if a lumbar puncture was carried out— in cerebrospinal fluid. A participant was defined as a malaria case if the Bioline Malaria Ag *P.f.* RDT was positive. All other participants were considered respectively gHAT negative or malaria negative. Statistical analyses were carried out in Graphpad Prism software version 10.0 (Boston, USA). The difference in age between individuals in Côte d’Ivoire and Guinea was assessed by an unpaired T test. Specificity of the RDT and laboratory tests was calculated with 95% Wilson Brown confidence intervals. Differences in fractions between groups (Côte d’Ivoire versus Guinea, or malaria positive versus malaria negative) were assessed by Chi square tests. Differences in specificity between 2 tests carried out on the same individuals were assessed by McNemar (Graphpad prism McNemar https://www.graphpad.com/quickcalcs/McNemar1.cfm). Agreement between test results was determined through calculating Cohen’s kappa coefficient (ƙ) (Graphpad prism https://www.graphpad.com/quickcalcs/kappa1/) [27].

## Results

### Demographic data

In the field, 1105 individuals were sequentially included and tested. Data from 10 individuals (9 from Guinea and 1 from Côte d’Ivoire) were retrospectively removed from the database since eight individuals were younger than 10 years and not eligible; one individual had been diagnosed and treated for gHAT previously and was not eligible; and one individual had a missing parasitology result due to a failing mAECT column, which could not be repeated. Out of the 1095 individuals retained for analysis, 576 originated from Côte d’Ivoire (52.6%, 284 from Bonon and 292 from Sinfra), and 519 from Guinea (47.4%, 185 from Ouassou and 334 from Khorira). Demographic characteristics of the study participants in Côte d’Ivoire and in Guinea are summarized in Table 5. In Côte d’Ivoire compared to Guinea, significantly more males participated and the mean participant age was higher, while the proportion of malaria patients was lower.

**Table 5:** Demographic characteristics of study participants in Côte d’Ivoire and in Guinea

**Variables**	**Total**	**Côte d’Ivoire**	**Guinea**	***P* value**
Number	1095	576	519	
Female (%)	617 (56.3%)	293 (50.9%)	324 (62.4%)	≤ 0.0001
Mean age (± standard deviation)	39 (± 18)	45 (± 17)	34 (± 17)	≤ 0.0001
Malaria positive (%)	278 (25.4%)	105 (18.2%)	173 (33.3%)	≤ 0.0001
≥ 1 gHAT screening test positive (%)	423 (38.6%)	268 (46.5%)	155 (29.9%)	≤ 0.0001
gHAT (%)	1 (0.09%)	0 (0.00%)	1 (0.19%)	

Differences in fractions between Côte d’Ivoire and Guinea were assessed by Chi square

### gHAT screening test results, parasitology and specificity

Overall, screening test seropositivity was 38.6% (423/1095) (Fig. 1), with significantly more gHAT screening test positive individuals in Côte d’Ivoire than in Guinea (Table 5). Among the 423 screening test positives that underwent mAECT, one gHAT patient was diagnosed in Guinea, with a trypanosome positive mAECT. This gHAT patient was positive for all gHAT screening tests and in all RDT test lines, and tested malaria positive. No lymph node puncture was carried out, nor any lumbar punctures. In the study, 1094 individuals, including 422 mAECT negative screening test positives, were therefore considered as not infected by gHAT (Fig. 1). Among these 422 individuals, 178 were positive in 1 screening test, 116 in 2 screening tests, 96 in 3 screening tests, 30 in 4 screening tests and 2 in all screening tests.

**Fig. 2:**
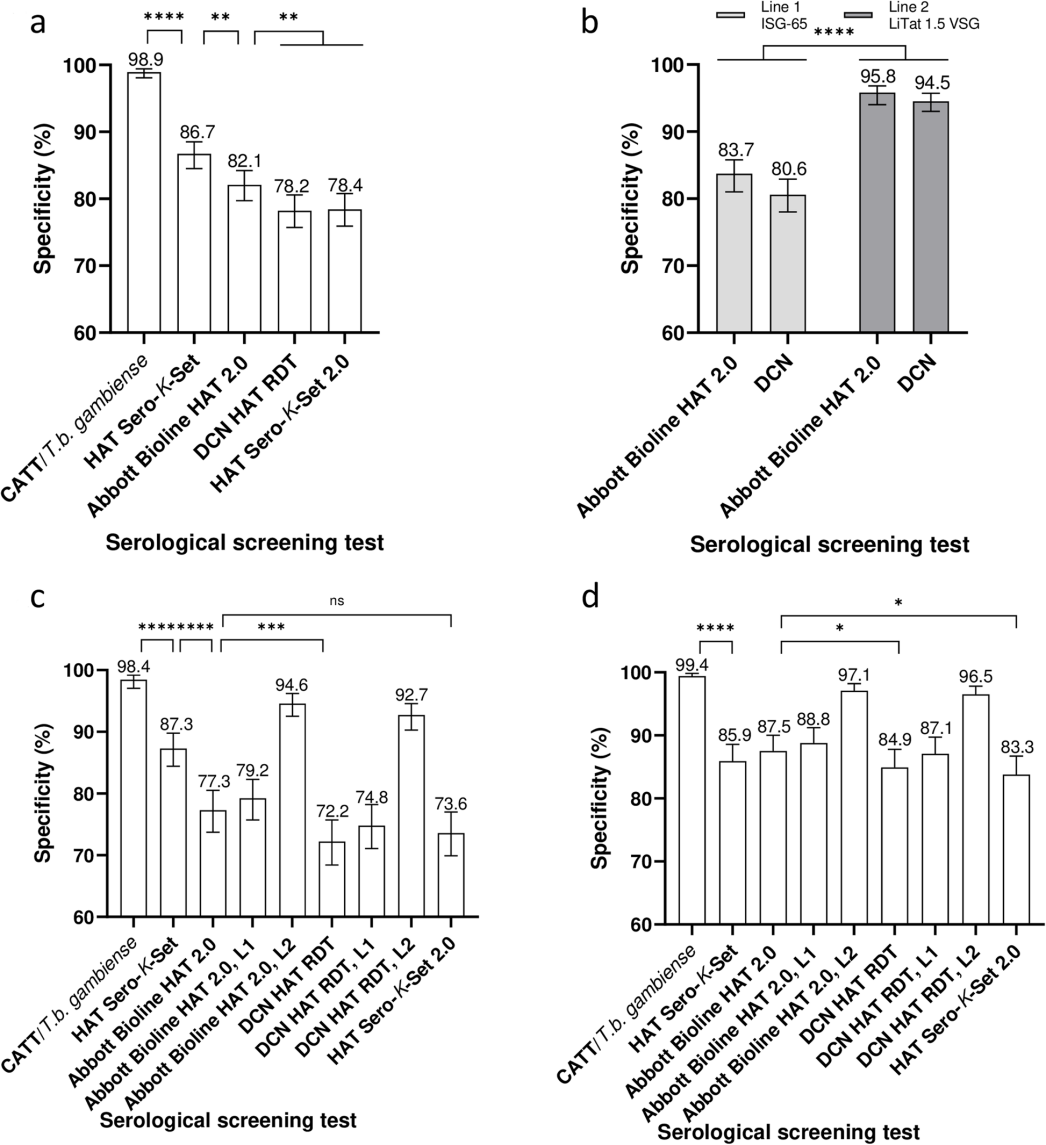
Specificity of serological screening tests to diagnose gambiense human African trypanosomiasis. Percent specificities with 95% confidence intervals. **a** of gHAT screening tests on all gHAT negative specimens (*n* = 1094); **b** of individual test lines in Abbott Bioline HAT 2.0 and DCN (*n* = 1094); **c** of gHAT screening tests and individual RDT test lines on HAT negative specimens from Côte d’Ivoire (*n* = 576); **d** of gHAT screening tests and individual RDT test lines on HAT negative specimens from Guinea (*n* = 518). Differences in specificity with ****P ≤ 0.0001, *** *P* ≤ 0.001, ** *P* ≤ 0.01 and * *P* ≤ 0.05. *L1* line 1; *L2* line 2

Details of the screening test specificities are shown in Additional file 1. Overall, the specificity of CATT (1082/1094, 98.9%, 95% *CI*: 98.1–99.4%) was significantly higher than the specificities of the HAT RDTs (*P* < 0.0001) (Fig. 2a). The specificity of the first-generation HAT Sero-*K*-Set RDT (948/1094, 86.7%, 95% *CI*: 84.5–88.5%) was significantly higher (*P* = 0.0014) than that of Abbott Bioline HAT 2.0 (898/1094, 82.1%, 95% *CI*: 79.7–84.2%) and significantly higher (*P* < 0.0001) than that of DCN HAT (856/1094, 78.2%, 95% *CI*: 75.7–80.6%) or HAT Sero-*K*-Set 2.0 (858/1094, 78.4%, 95% *CI*: 75.9–80.8%). Abbott Bioline HAT 2.0 was significantly more specific than DCN HAT RDT and HAT Sero-*K*-Set 2.0 (*P* < 0.0001 and 0.01 respectively). Only between Abbott Bioline HAT 2.0 and DCN HAT RDT test agreement was substantial (ƙ = 0.7), for all other test combinations it was slight to fair (ƙ < 0.39).

In Côte d’Ivoire (Fig. 2c,d), second generation RDT specificities were significantly lower than in Guinea (*P* < 0.0001), but this was not the case for CATT and HAT Sero-*K*-Set (Additional file 1). The difference between Abbott Bioline HAT 2.0 and HAT Sero-K-Set 2.0 was not significant (*P* = 0.1) in Côte d’Ivoire. In Guinea, the difference between HAT Sero-K-Set and the 2nd generation RDTs was not significant. Abbott Bioline HAT 2.0 was slightly more specific (*P* = 0.04) than DCN HAT RDT or HAT Sero-*K*-Set 2.0.

Abbott Bioline HAT 2.0 and DCN HAT RDT both consist of 2 individual test lines with the same antigens, ISG65 in line 1 and LiTat 1.5 VSG in line 2. For both tests the specificity of test line 1 with specificities of respectively 83.7% and 80.6% was significantly lower (*P* < 0.0001) than that of test line 2 with specificities of 95.8% and 94.5% (Fig. 2b). Similar *P* values were observed when the datasets of Côte d’Ivoire or Guinea were considered separately.

### RDT test line intensities

**Fig. 3:**
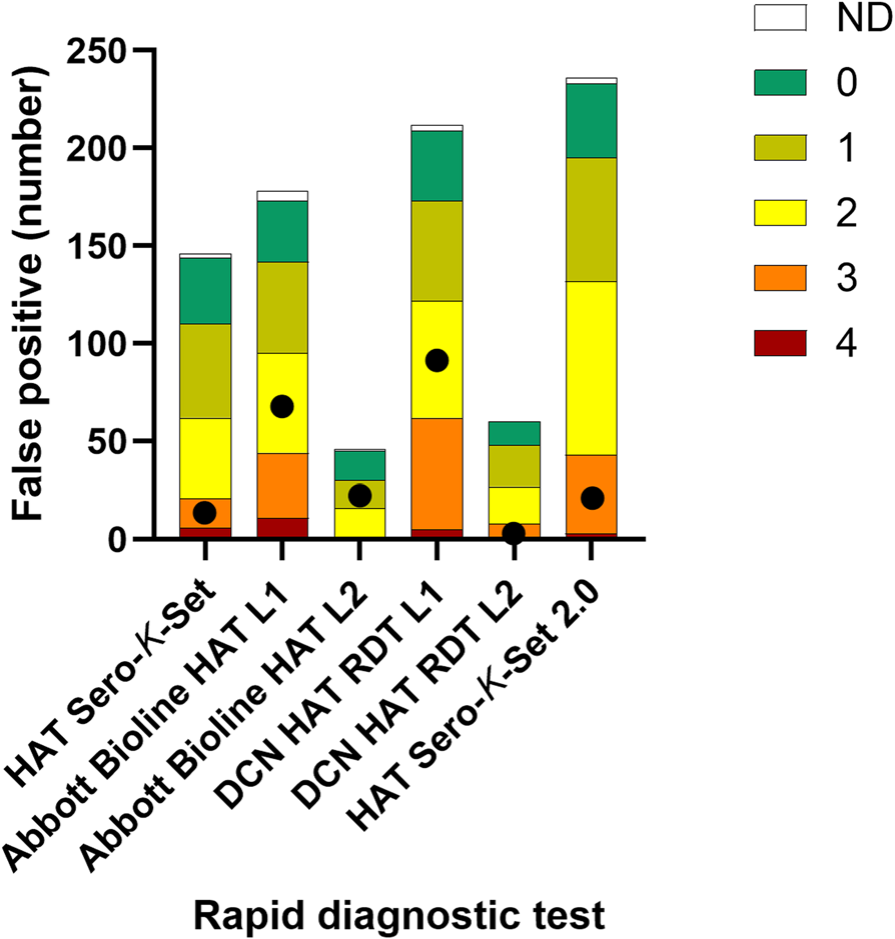
Retrospective scoring of line intensities in pictures taken from false positive rapid diagnostic tests. Line intensities observed on the picture were compared to intensities on a reference colour chart [25]: 0 negative; 1 doubtful; 2 weakly positive; 3 medium positive; 4 strongly positive. ● Line intensity observed in the HAT positive patient. *L1* line 1; *L2* line 2. *ND* not done

For the 423 seropositives, 414 pictures were available for a posteriori reading of line intensities of all the RDTs of each individual. Figure 3 illustrates the line intensities of the false positive test lines in the field. Clearly positive scores of ≥ 2 were observed in respectively 62/144 (HAT Sero-*K*-Set), 95/173 and 16/45 (Abbott Bioline HAT 2.0 lines 1 and 2, respectively), 122/209 and 27/60 (DCN HAT RDT) and 132/233 (HAT Sero-*K*-Set 2.0). On the 414 pictures that were available of the seropositive group, also tests and test lines that had been scored negative in the field were read. Retrospective reading of test line intensities of these field negative lines scored ≥ 2 in respectively 0/269 (HAT Sero-*K*-Set), 0/240 and 0/368 (Abbott Bioline 2.0 line 1 and 2), 2/204 and 0/353 (DCN HAT RDT) and 3/180 (HAT Sero-*K*-Set 2.0) indicating that probably only a few weak positive test lines had been missed in the field. In the confirmed HAT patient, the line intensities were respectively 3, 2, 1, 2, 3 and 3.

### Specificity of gHAT screening tests in function of malaria status

**Fig. 4:**
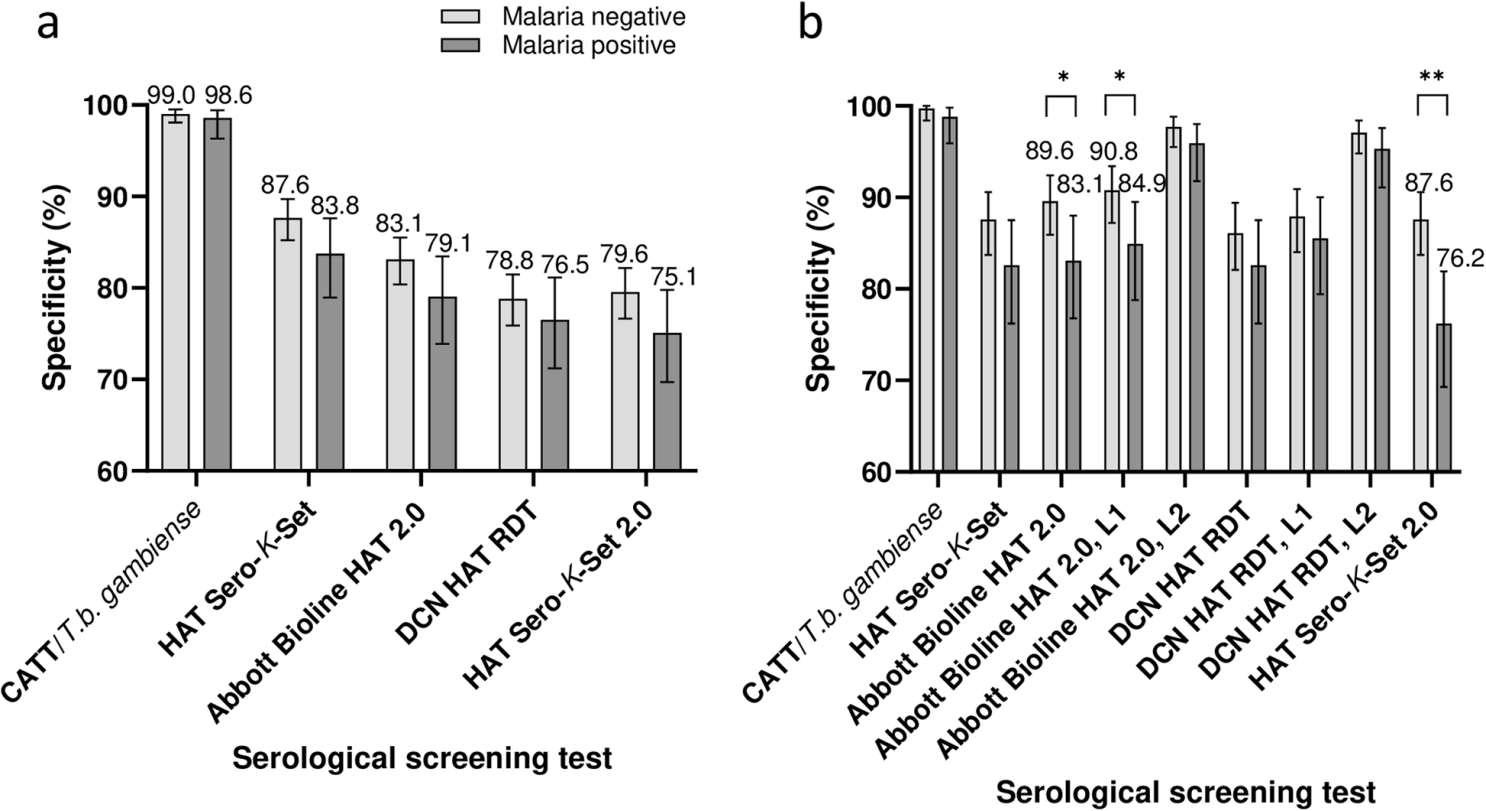
Specificity of serological screening tests for diagnosis of gHAT and of rapid diagnostic test individual lines in function of malaria infection. Percent specificities with 95% confidence intervals. a. for all gHAT negative specimens (*n* = 1094, malaria positive *n* = 277, malaria negative *n* = 817); b. of gHAT screening tests and individual RDT test lines on HAT negative specimens from Guinea only (*n* = 518, malaria positive *n* = 173, malaria negative *n* = 346). *L1* line 1; *L2* line 2. * *P* < 0.05; ** *P* ≤ 0.001

Although for all screening tests and test lines the specificity in the malaria positive group (*n* = 277) was lower than in the malaria negative group (*n* = 817), differences were not significant (*P* values of 0.08–0.6, Fig. 4, Additional file 1). The same was observed in the Côte d’Ivoire subgroup (*P* values of 0.1–1). However, in Guinea, significant differences in specificity were observed using the Abbott Bioline HAT 2.0 (RDT specificity of 89.6% in malaria negatives versus 83.1% in malaria positives, *P* = 0.03), in particular for test line 1 (90.8% versus 84.9% in malaria negatives versus positives, *P* = 0.03), and for HAT Sero-*K*-Set 2.0 (87.6% versus 76.2% in malaria negatives versus positives, *P* = 0.0006).

### Reference laboratory test results

Dried blood spots were available from 400/423 screening test seropositives, blood on DNA/RNA Shield buffer from 303/423 screening test seropositives, including the gHAT patient.

The results obtained in the *g*-iELISAs were not exploitable. In all 10 plates, the percent positivity of the positive control was below the threshold value, while in respectively 4 and 1 plates, the optical density of the negative control for respectively LiTat 1.3 and LiTat 1.5 VSG was below the threshold.

Eight out of 400 seropositives tested positive for the other immunological reference laboratory tests. The gHAT patient was trypanolysis LiTat 1.3 and trypanolysis LiTat 1.5 positive. Among the 399 non-confirmed screening test seropositives, 5 tested positive in ELISA/*T.b. gambiense*, 1 in trypanolysis on LiTat 1.3 and one in trypanolysis on LiTat 1.5, but no individual was positive in more than 1 test. Trypanolysis on LiTat 1.6 remained negative in all specimens. Test specificities are summarized in Table 6.

**Table 6:** Specificity of immunological and molecular reference laboratory tests in non-confirmed gHAT screening test seropositive subjects

	***n*/*N***	**Specificity (%)**	**95% *CI***
Indirect ELISA/*T.b. gambiense*	394/399	98.7	97.1**–**99.5
Trypanolysis			
LiTat 1.3^a^	398/399	99.7	98.6**–**100
LiTat 1.5^a^	398/399	99.7	98.6**–**100
LiTat 1.6	399/399	100	99.0**–**100
3 VATs in parallel^a^	397/399	99.5	98.2**–**99.9
PCR			
TBRN3^a^	287/302	95.0	92.0**–**97.0
18S	299/302	99.0	97.1**–**99.7
TgsGP	302/302	100	98.7**–**100
SHERLOCK			
18S Tids^a^	281/302	93.0	95.4**–**89.6
7SL *Zoon*	301/302	99.7	98.1**–**100
TgsGP	302/302	100	98.7**–**100
*Trypanozoon*-S^2^-RT-qPCR			
18S^a^	300/302	99.3	97.6**–**99.9
177T^a^	297/302	98.3	96.2**–**99.3
GPI-PLC	302/302	100	98.7**–**100
TgsGP	302/302	100	98.7**–**100
4 targets in parallel^a^	297/302	98.3	96.2**–**99.3
RT-qPCR			
DT8	302/302	100	98.7**–**100
DT9	299/302	99.0	97.1**–**99.7
TgsGP	302/302	100	98.7**–**100

*VAT* Variable antigen type, *95% CI* 95% confidence interval

^a^Positive result for the gHAT patient

All 303/303 specimens passed the SHERLOCK-RP quality control. In total 38/303 screening test seropositives were positive in ≥ 1 molecular reference laboratory test. The gHAT patient was positive for 4 molecular reference laboratory tests: SHERLOCK 18S Tids, Trypanozoon-S^2^-RT-qPCR 18S2, Trypanozoon-S^2^-RT-qPCR 177T, and TBRN3 PCR. Among the 37 other molecular reference laboratory test positive specimens that were not confirmed by parasitological examination, 2 specimens were positive in 3 molecular reference laboratory tests (*Trypanozoon*-S^2^-RT-qPCR 18S2, *Trypanozoon*-S^2^-RT-qPCR 177T, and TBRN3 PCR); 9 specimens were positive in 2 molecular tests and 26 specimens in 1 test only. The specificities of the molecular reference laboratory tests are listed in Table 6. Whatever the format, all tests targeting the TgsGP gene were 100% specific, as were *Trypanozoon*-S^2^-RT-qPCR-GPI-PLC and RT-qPCR DT8. SHERLOCK 18S Tids (with a cut-off FC > 5) and TBRN3 PCR were significantly less specific than the other molecular tests (*P* < 0.003 and *P* < 0.01 respectively).

Despite the high specificity of most of the 17 reference laboratory tests (not considering *g*-iELISA), among the 44 positives, few individuals showed combined positivity. The gHAT patient tested positive in 2 immunological and 4 molecular reference laboratory tests. Of the 2 individuals positive in 3 molecular reference laboratory tests, one was also positive in indirect ELISA/*T.b. gambiense*. This person was clearly positive in all 5 serological screening tests with line intensity scores of 2–4 and underwent repeated parasitological examinations, but could not be confirmed. No other subjects combined positivity in immunological and molecular reference laboratory tests. The other individual with 3 molecular reference laboratory tests positive, was positive only in the HAT Sero-*K*-Set screening test (line intensity score of 2). The remaining 41 individuals were either positive in 1 immunological reference laboratory test only (*n* = 6), in maximum 2 molecular reference laboratory tests (*n* = 9) or in 1 molecular reference laboratory test only *n* = 26).

## Discussion

For serological screening for gHAT, the specificity of the CATT test of 98.9% was significantly higher than the specificities of the RDTs, which ranged only from 78.2% to 86.7% and were far below the minimal specificity required for identifying individuals for treatment according to the WHO target product profile [6]. The specificities of the immunological reference laboratory tests trypanolysis and indirect ELISA/*T.b. gambiense* were ≥ 98.7%, while specificities of the molecular reference laboratory tests SHERLOCK, *Trypanozoon*-S2-RT-qPCR, RT-qPCR and PCR were 93.0–100%, depending on the target. Despite this high specificity, the reference laboratory test positive results were dispersed and lacked coherence among the tests.

An important strength of the present diagnostic study is the battery of tests carried out. Indeed, all commercialized and prototype gHAT screening tests were included, as well as all immunological and molecular reference laboratory tests that are actually proposed for gHAT reference testing of screening test seropositives.

Some limitations in the set-up and practical implementation of the diagnostic trial should be highlighted. First, we assumed that sensitivity of screening would be 100% by applying 5 gHAT screening tests in parallel. Individuals negative in all 5 screening tests were not examined microscopically, as this would have overloaded the mobile team and diluted HAT control efforts instead of focussing on individuals at highest suspicion. Furthermore, a parasitology composite reference standard was used, but in practice, as lymph node or cerebrospinal fluid examinations were not relevant, parasitology relied exclusively on the mini-anion exchange centrifugation technique, which is around 90% sensitive [28]. On the other hand, few gHAT cases could be expected for the simple reason that gHAT prevalence in all study areas was probably well below 1/10,000 [3, 4]. With such a low prevalence, assessment of the test sensitivity was not feasible. Following gHAT elimination as a public health problem in Africa [17], all future prospective clinical performance studies will be confronted with this limitation, in whatever gHAT endemic area they are carried out, underlining the importance of specimen banks for test evaluation [29]. Limitations in practical implementation of the study, include the use of heparinized venous blood, as the volume of finger prick blood would have been insufficient for all gHAT screening tests. Furthermore, because of the unanticipated high seroprevalence, the material for preparing DBS and blood in RNA/DNA Shield buffer was insufficient. Although extra material could be provided within days, this resulted in incomplete collection of seropositive specimens for the reference laboratory tests. The study employed a single nucleic acid extraction technique on a volume of blood smaller than what is used in mAECT. As with parasitological tests, the sensitivity of molecular assays is likely to improve when larger volumes of specimens can be extracted.

The high seroprevalence was mainly caused by the unexpected low specificity of the RDTs, while the specificity of CATT was in line with previous observations [9, 10, 25, 30, 31]. For the prototype gHAT RDTs DCN HAT RDT and HAT Sero-*K*-Set 2.0, prospective studies have not been previously carried out. A prospective diagnostic trial on a prototype Abbott Bioline HAT 2.0 RDT (under the name SD Bioline HAT 2.0) was carried out in Democratic Republic of Congo (DRC) and reported specificities of respectively 99.1% and 93.7% in passive and in active screening. During door to door screening in Guinea in 2021, positivity rates between 3.7 and 6% were observed with the Abbott Bioline HAT 2.0 RDT [32].

The first prospective clinical performance study of the first generation HAT Sero-*K*-Set in DRC demonstrated a specificity of 98.6% [9], based on a mix of active and passive recruitment. Next, specificities of 97.0–97.8% were observed in prospective trials in DRC, Côte d’Ivoire and Guinea, based on passive screening [13, 33, 34]. More recently, during active screening in Burkina Faso, 89.1% specificity was obtained [35], while in door-to-door screening in Guinea, positivity rates ranged 1.9–9.4% [32]. The RDT specificities observed in the present study were all below previously reported values and well below the 95% specificity requirement of the WHO target product profile [6]. Interestingly, although there were intercountry variations, low RDT specificity was not only observed in the present study by the two independent teams of Côte d’Ivoire and Guinea but was also reported in a similar prospective trial which was carried out in parallel in DRC and followed a similar study protocol (NCT05637632, Tablado-Alonso, personal communication). The reason for the observed low specificities is not entirely clear. Diagnostic test evaluation in the actual SpeSerTryp study was carried out through active screening by specialized gHAT mobile teams. This way of working could result in more accurate reading of the RDT result, than by staff doing routine laboratory work in passive screening. In addition, working in open air could influence test conditions, through improved visibility of weak test lines in higher light intensities. Although roughly half of the false positive test lines were retrospectively quantified as doubtful or negative on the picture, the other half was clearly positive and could hardly be missed. Increased evaporation in open air could also play a role. Although from the present results, we cannot exclude that malaria might lower gHAT RDT specificity, as previously observed in Guinea [32], this does not seem to be the main reason for the observed low RDT specificities. However, both in the Abbott Bioline HAT 2.0 and the DCN HAT RDT, the specificity of line 1, ISG65, was significantly lower than that of line 2, LiTat 1.5 VSG, which consistently showed around 95% specificity. Although it cannot be excluded that the first test line which is encountered by the blood specimen might accumulate nearly all non-specific reactions, our results suggest that false positive reactions with recombinant ISG65 expressed in *Escherichia coli* is a major reason for low specificity in these RDTs. This hypothesis cannot be extrapolated to HAT Sero-*K*-Set 2.0, which also has recombinant ISG65 in its test line, as it is expressed in insect cells and mixed with recombinant LiTat 1.3 and 1.5 VSG. Compared to the first generation RDTs, inclusion of recombinant ISG65 in 2nd generation RDTs was based on encouraging results of phase 2 evaluations with research prototype RDTs including recombinant ISG65 [36, 37]. However, one of those studies already highlighted a potential lower specificity of ISG65 alone or in combination with recombinant LiTat 1.5 VSG compared to native VSGs [37].

Among the immunological reference laboratory tests, the specificity values obtained for trypanolysis on DBS are in line with previously reported values of 92.9–100% [13, 34, 35], although LiTat 1.6 is not often included in the trypanolysis test battery. For indirect ELISA/*T.b. gambiense* as well, specificity was in the range of the 95.3–99.8% values reported in the literature [13, 20, 34, 35]. The indirect ELISA/*T.b. gambiense* was negative for the confirmed gHAT case, while trypanolysis was negative in an indirect ELISA/*T.b. gambiense* positive specimen positive in the molecular tests as well. This could be due to the use of DBS, which is less sensitive than plasma for trypanolysis, and has moderate sensitivity in indirect ELISA/*T.b. gambiense* as well [19, 34]. Overall, both trypanolysis and indirect ELISA/*T.b. gambiense* seem to comply with the desirable specificity stipulated in the WHO target product profile for an individual test to assess infection in low prevalence settings [11]. For the *g*-iELISA, failure of the internal controls to fall within the acceptance criteria defined by the manufacturer might be explained by limited stability of the VSG epitopes reacting with the monoclonal antibodies in the kit [14].

Among the molecular tests, the specificities observed with respectively the SHERLOCK 18S Tids assay and TBRN3 PCR were lower than for the other tests and lower than the minimal 95% requirement of the target product profile [11]. For SHERLOCK 18S Tids, this could be due to the low positivity cut-off value used in this study (FC > 5) and / or because of target specificity, being the Trypanosomatid family, which might, besides *Trypanozoon* parasites, detect other pathogens of the *Trypanosoma* and *Leishmania* genera. A number of animal trypanosomes are circulating in the study areas, some of which could incidentally infect humans [38], and Guinea and Côte d’Ivoire are considered endemic for leishmaniasis [39–42]. Potential issues with the specificity of the TBRN3 PCR targeting the TBR tandem repeat DNA sequence in seropositive individuals have already been highlighted in the past [43, 44], although the primers used in those studies were slightly different [45]. The recently developed *Trypanozoon*-S^2^-RT-qPCR 177T [16] also targets TBR DNA, but does not seem to suffer from the specificity issues observed with TBRN3 PCR. The *Trypanozoon*-S^2^-RT-qPCR 177T is, in combination with *Trypanozoon*-S^2^-RT-qPCR 18S2, already routinely implemented in DRC for examining seropositive individuals. With 98.3–99.3% specificity depending on serial or parallel interpretation of the *Trypanozoon*-S^2^-RT-qPCR 177T and 18S2 results, *Trypanozoon*-S^2^-RT-qPCR 177T and 18S2 comply to the target product profile desired specificity, and both targets also detected the gHAT case. Further studies on *Trypanozoon*-S^2^-RT-qPCR 177T and 18S2 diagnostic performances are therefore warranted. The SHERLOCK 7SL *Zoon* assay [15] appeared to be highly specific in the present study but did not pick up the gHAT case. However, it also merits further evaluation as other studies, although using a different detection format, highlighted the promising diagnostic accuracy of 7SL small RNA detection for *Trypanozoon* detection in animals [46, 47]. Specificity of 18S PCR is in line with a previous report [22] and although it did not detect the gHAT patient in the present study, its sensitivity has been previously estimated to be sufficient [22, 28]. All test formats targeting TgsGP had 100% specificity but this target, which is a single copy gene, is already known to have low analytical and diagnostic sensitivity [23]. So far, no previous diagnostic accuracy evaluations have been published for the other test formats. Like TgsGP, the diagnostic specificity of the RT-qPCR DT8 and DT9 seems quite elevated, but diagnostic sensitivity is expected to be low.

Overall, despite the high specificity of most reference laboratory tests, the incoherence of the test results within the seropositive group remains problematic [43], and is an issue to be solved, not only for individual diagnosis of gHAT suspects, but also for country verification of zero transmission, for which further reference laboratory examinations of all screening test seropositives is recommended [48].

The results of the present study have important implications for practice. The first outcome is the choice of a suitable test for screening of the population at risk. Due to its excellent specificity, which is significantly higher than that of all gHAT RDTs, CATT remains the preferred option for mass population screening, whenever possible. For individual and small-scale screening, or whenever CATT is not feasible, RDTs can be used, but it should be kept in mind that their specificity is low and might overload the parasitological confirmation work. Reading only the LiTat 1.5 test line (line 2) in Abbott Bioline HAT 2.0 or the DCN HAT RDT would increase specificity but might result in insufficient sensitivity and implies a risk of missing true gHAT cases. While waiting for improved RDTs, a solution could be to perform Abbott Bioline HAT 2.0 first, and on Abbott Bioline HAT 2.0 positives, to perform HAT-Sero-*K*-Set next (in series combination of both tests), as presently done by the Guinean gHAT control program [32], which in the present study had a specificity of 95.1% (1040/1094, 95% *CI*: 93.6–96.2%). Looking at the near future, the actually commercialized RDTs, HAT Sero-*K*-Set or Abbott Bioline HAT 2.0, are unsuited for implementing a “screen and treat” strategy once safety of acoziborole for treatment of seropositive suspects has been sufficiently demonstrated [49], as their use could lead to massive overtreatment of up to 17.9% of the tested population, while the gHAT prevalence is as low as 1/10,000 or less.

Based on the present results, giving recommendations on reference laboratory tests to be used to confirm seropositive individuals is tricky, as reference laboratory test results were insufficiently coherent to reliably discriminate false screening test positives from *T.b. gambiense* infected individuals.

## Conclusions

Specificity values of gHAT RDTs were lower than the 95% stipulated by the WHO target product profile. Antigen production for CATT and the 1st generation RDTs relies on animal infection with virulent human infectious *T.b. gambiense* parasites, raising ethical and safety concerns and therefore being unsustainable. Improved 2nd generation RDTs need to be developed, in particular in view of the future implementation of “screen & treat” to accelerate gHAT elimination. Taking into account the proven outstanding diagnostic accuracy of the 3 antigens actually in use [50], and the lack of new better alternatives, the choice of including in particular ISG65 in the RDT test lines needs to be reconsidered and new recombinant expression systems should be explored. Also, during test development, more attention should be given to reducing non-specific reactions by optimizing test conditions. Considering the laboratory tests, further comparative evaluation, especially of the molecular test performances, is an urgent requirement.

### Supplementary Information

Additional file 1. Specificity of gHAT screening tests and individual RDT test lines in the complete test group, by country and by malaria status. Differences in specificity between Côte d’Ivoire and Guinea, or between malaria positive and malaria negative groups were assessed by Chi square.

## Data Availability

The public sharing of personal health data is subject to the General Data Protection Regulation. The health data underlying the findings described in the manuscript can therefore not be made public. Metadata are publicly available in IRD’s institutional repository DataSuds via “Lejon, Veerle; Bieler, Sylvain; Camara, Oumou; N’Djetchi, Kassi Martial; Jamonneau, Vincent; Bart, Jean-Mathieu; Bucheton, Bruno; Solano, Philippe; Rotureau, Brice; Kaba, Dramane; N'Dri, Louis; Koffi, Mathurin; Bessell, Paul, 2022, “Prospective evaluation of the specificity of serological tests for Human African Trypanosomiasis: documentation and data”, 10.23708/APDHAG [51]. The datasets generated and analysed in the present manuscript will be made available to qualified researchers upon request and after signing a confidentiality agreement. Data requests may be sent to the Institut de Recherche pour le Développement (IRD) data administrator (data@ird.fr).

## Abbreviations

CATT: Card Agglutination Test for Trypanosomiasis
*CI*: Confidence interval
DBS: Dried blood spot
DRC: Democratic Republic of the Congo
ELISA: Enzyme-linked immunosorbent assay
FC: Fold-change over the NTC background fluorescence
gHAT: Gambiense Human African trypanosomiasis
*g*-iELISA: Gambiense inhibition ELISA
GPI-PLC: Glycosylphosphatidylinositol-specific phospholipase C
ISG65: Invariable surface glycoprotein 65
IVT: In vitro transcription
ƙ: Cohen’s kappa coefficient
LiTat: Lille Trypanosome antigen type
mAECT: Mini anion exchange centrifugation technique
NTC: Negative template control
PCR: Polymerase chain reaction
RDT: Rapid diagnostic test
RP: Human RNase P POP7
RPA: Recombinase polymerase amplification
RT-qPCR: Reverse transcription-quantitative polymerase chain reaction
SHERLOCK: Specific High Sensitivity Enzymatic Reporter unlocking
SpeSerTryp: Prospective evaluation of the SPEcificity of SERological tests for human African TRYPanosomiasis in Côte d’Ivoire and Guinea
TBR: *Trypanosoma brucei repeat*
*T.b. gambiense*: *Trypanosoma brucei gambiense*
TgsGP: *Trypanosoma brucei gambiense* Specific glycoprotein
TNA: Total Nucleic acid
VAT: Variable Antigen Type
VSG: Variable Surface Glycoprotein
WHO: World Health Organization
