# Supplementary material for: Specificity of serological screening tests and reference laboratory tests to diagnose gambiense human African trypanosomiasis: a prospective clinical performance study
Source: Infect Dis Poverty. 2024 Jul 8;13:53. doi: 10.1186/s40249-024-01220-5 (PMC11229219; doi:10.1186/s40249-024-01220-5)
Supplement: Supplementary file 1 — Additional file 1. Specificity of gHAT screening tests and individual RDT test lines in the complete test group, by country and by malaria status. Differences in specificity between Côte d’Ivoire and Guinea, or between malaria positive and malaria negative groups were assessed by Chi square. [file 40249_2024_1220_MOESM1_ESM.docx]

Additional information file 1: Specificity of gHAT screening tests and individual RDT test lines in the complete test group, by country and by malaria status. Differences in specificity between Côte d’Ivoire and Guinea, or between malaria positive and malaria negative groups were assessed by Chi square.

|  | **Côte d’Ivoire + Guinea** | | | ***P* *CI* versus GN** | **Côte d’Ivoire (CI)** | | | **Guinea (GN)** | | |
| --- | --- | --- | --- | --- | --- | --- | --- | --- | --- | --- |
|  | ***n/N*** | **% Specificity**  **(95% *CI*)** | ***P* Malaria**  **Neg versus pos** |  | ***n/N*** | **% Specificity**  **(95% *CI*)** | ***P* Malaria**  **Neg versus pos** | ***n/N*** | **% Specificity**  **(95% *CI*)** | ***P* Malaria**  **Neg versus pos** |
| CATT | | | | | | | | | | |
| Total | 1082/1094 | 98.9 (98.1**–**99.4) |  | 0.1 | 567/576 | 98.4 (97.1–99.2) |  | 515/518 | 99.4 (98.3–99.8) |  |
| Malaria negative | 809/817 | 99.0 (98.1**–**99.5) | 0.3 |  | 464/471 | 98.5 (97.0–99.3) | 0.8 | 345/346 | 99.7 (98.4–100.0) | 0.08 |
| Malaria positive | 273/277 | 98.6 (99.4**–**96.3) |  |  | 103/105 | 98.1 (93.3–99.7) |  | 170/172 | 98.8 (95.9–99.8) |  |
| HAT Sero-*K*-Set | | | | | | | | | | |
| Total | 948/1094 | 86.7 (84.5**–**88.5) |  | 0.5 | 503/576 | 87.3 (84.4–89.8) |  | 445/518 | 85.9 (82.6–88.6) |  |
| Malaria negative | 716/817 | 87.6 (85.2**–**89.7) | 0.08 |  | 413/471 | 87.7 (84.4–90.4) | 0.6 | 303/346 | 87.6 (83.7–90.6) | 0.09 |
| Malaria positive | 232/277 | 83.8 (79.0**–**87.6) |  |  | 90/105 | 85.7 (77.8–91.1) |  | 142/172 | 82.6 (76.2–87.5) |  |
| Abbott Bioline HAT 2.0 | | | | | | | | | | |
| Total | 898/1094 | 82.1 (79.7**–**84.2) |  | < 0.0001 | 445/576 | 77.3 (73.7–80.5) |  | 453/518 | 87.5 (84.3–90.0) |  |
| Malaria negative | 679/817 | 83.1 (80.4**–**85.5) | 0.1 |  | 369/471 | 78.3 (74.4–81.8) | 0.2 | 310/346 | 89.6 (85.9–92.4) | 0.03 |
| Malaria positive | 219/277 | 79.1 (73.9**–**83.4) |  |  | 76/105 | 72.4 (63.2–80.0) |  | 143/173 | 83.1 (76.8–88.0) |  |
| Abbott Bioline HAT 2.0, line 1 | | | | | | | | | | |
| Total | 916/1094 | 83.7 (81.4**–**85.8) |  | < 0.0001 | 456/576 | 79.2 (75.7–82.3) |  | 460/518 | 88.8 (85.8–91.2) |  |
| Malaria negative | 692/817 | 84.7 (82.1–87.0) | 0.1 |  | 378/471 | 80.3 (76.4–83.6) | 0.2 | 314/346 | 90.8 (87.2–93.4) | 0.03 |
| Malaria positive | 224/277 | 80.9 (75.8–85.1) |  |  | 78/105 | 74.3 (65.2–81.7) |  | 146/172 | 84.9 (78.8–89.5 |  |
| Abbott Bioline HAT 2.0, line 2 | | | | | | | | | | |
| Total | 1048/1094 | 95.8 (94.4–96.8) |  | 0.04 | 545/576 | 94.6 (92.5–96.2) |  | 503/518 | 97.1 (95.3–98.2) |  |
| Malaria negative | 785/817 | 96.1 (94.5–97.2) | 0.3 |  | 447/471 | 94.9 (92.5–96.6) | 0.5 | 338/346 | 97.7 (95.5–98.8) | 0.2 |
| Malaria positive | 263/277 | 94.9 (91.7–97.0) |  |  | 98/105 | 93.3 (86.9–96.7) |  | 165/172 | 95.9 (91.8–98.0) |  |
| DCN HAT RDT | | | | | | | | | | |
| Total | 856/1094 | 78.2 (75.7–80.6) |  | < 0.0001 | 416/576 | 72.2 (68.4–75.7) |  | 440/518 | 84.9 (81.6–87.8) |  |
| Malaria negative | 644/817 | 78.8 (75.9–81.5) | 0.4 |  | 346/471 | 73.5 (69.3–77.2) | 0.2 | 298/346 | 86.1 (82.1–89.4) | 0.2 |
| Malaria positive | 212/277 | 76.5 (71.2–81.1) |  |  | 70/105 | 66.7 (57.2–75.0) |  | 142/172 | 82.6 (76.2–87.5) |  |
| DCN HAT RDT, line 1 | | | | | | | | | | |
| Total | 882/1094 | 80.6 (78.2–82.9) |  | < 0.0001 | 431/576 | 74.8 (71.1–78.2) |  | 451/518 | 87.1 (83.9–89.7) |  |
| Malaria negative | 663/817 | 81.2 (78.3–83.7) | 0.4 |  | 359/471 | 76.2 (72.2–79.8) | 0.1 | 304/346 | 87.9 (84.0–90.9) | 0.4 |
| Malaria positive | 219/277 | 79.1 (73.9–83.4) |  |  | 72/105 | 68.6 (59.2–76.7) |  | 147/172 | 85.5 (79.4–90.0) |  |
| DCN HAT RDT, line 2 | | | | | | | | | | |
| Total | 1034/1094 | 94.5 (93.0–95.7) |  | 0.006 | 534/576 | 92.7 (90.3–94.6) |  | 500/518 | 96.5 (94.6–97.8) |  |
| Malaria negative | 773/817 | 94.6 (92.8–96.0) | 0.6 |  | 437/471 | 92.8 (90.1–94.8) | 0.9 | 336/346 | 97.1 (94.8–98.4) | 0.2 |
| Malaria positive | 261/277 | 94.2 (90.8–96.4) |  |  | 97/105 | 92.4 (85.7–96.1) |  | 164/172 | 95.3 (91.1–97.6) |  |
| HAT Sero-*K*-Set 2.0 | | | | | | | | | | |
| Total | 858/1094 | 78.4 (75.9–80.8) |  | < 0.0001 | 424/576 | 73.6 (69.9–77.0) |  | 434/518 | 83.8 (80.4–86.7) |  |
| Malaria negative | 650/817 | 79.6 (76.7–82.2) | 0.1 |  | 347/471 | 73.7 (69.5–77.4) | 0.9 | 303/346 | 87.6 (83.7–90.6) | 0.0006 |
| Malaria positive | 208/277 | 75.1 (69.7–79.8) |  |  | 77/105 | 73.3 (64.2–80.9) |  | 131/172 | 76.2 (69.3–81.9) |  |
| Serial: Abbott Bioline HAT 2.0 followed by HAT Sero-*K*-Set | | | | | | | | | | |
| all | 1040/1094 | 95.1 (93.6–96.2) |  | 0.4 | 544/576 | 94.4 (92.3–96.0) |  | 496/518 | 95.8 (93.7–97.2) |  |
| Malaria negative | 779/817 | 95.3 (93.7–96.6) | 0.3 |  | 445/471 | 94.5 (92.0–96.2) | 1 | 334/346 | 96.5 (94.0–98.0) | 0.2 |
| Malaria positive | 261/277 | 93.9 (90.4–96.1) |  |  | 99/105 | 94.3 (88.1–97.4) |  | 162/172 | 94.2 (89.6–96.8) |  |
